# Synergistic Interactions Between Inulin-Type Fructans and Plant Polyphenols: Implications for Antioxidant Activity, Bioavailability, and Functional Food Development

**DOI:** 10.3390/antiox15070788

**Published:** 2026-06-24

**Authors:** Anca Daniela Raiciu, Mihaela Carmen Eremia, Amalia Stefaniu

**Affiliations:** 1 Pharmacognosy, Phytochemistry, Phytotherapy Department, Pharmacy Faculty, “Titu Maiorescu” University, Gheorghe Şincai Street No. 16, District 4, 040314 Bucharest, Romania; 2 PLANTA ROMANICA Association, George Enescu Street No. 27-29, District 1, 010303 Bucharest, Romania; 3 Hofigal Export-Import S.A., Intrarea Serelor Street No. 2, District 4, 042124 Bucharest, Romania; 4 National Institute for Chemical Pharmaceutical Research and Development—ICCF, 112 Vitan Street, District 3, 031299 Bucharest, Romania; mihaelaceremia@yahoo.com (M.C.E.); astefaniu@gmail.com (A.S.)

**Keywords:** inulin, polyphenols, antioxidant activity, prebiotics, functional foods, bioavailability, plant extracts

## Abstract

Inulin-type fructans are widely recognized as functional polysaccharides with prebiotic properties, while plant polyphenols represent one of the most important classes of natural antioxidants. Increasing evidence demonstrates that interactions between dietary fibers such as inulin and phenolic compounds significantly influence antioxidant capacity, bioavailability, and physiological activity. The present review integrates recent advances regarding the chemical structure of inulin, extraction sources, molecular interactions with polyphenols, and implications for antioxidant activity in functional foods and nutraceuticals. Experimental studies indicate correlations between inulin concentration and antioxidant parameters such as DPPH, FRAP, SOD and CAT activities. Furthermore, physicochemical interactions between cell wall polysaccharides and polyphenols influence the stability, release kinetics and bioefficacy of antioxidant compounds. These findings support the potential development of optimized functional formulations combining inulin-rich plant extracts with polyphenol sources for improved health benefits. The literature was identified through searches of PubMed, Scopus and Web of Science databases (2000–2026).

## 1. Introduction

Dietary fibers and plant polyphenols represent major classes of bioactive compounds widely distributed in medicinal and aromatic plants (MAPs), contributing significantly to antioxidant defense mechanisms, metabolic regulation and prevention of chronic diseases. In recent decades, increasing scientific interest has focused on understanding the synergistic interactions between plant polysaccharides and phenolic compounds, due to their combined effects on oxidative stress modulation, gut microbiota balance, and bioavailability of phytochemicals [1,2,3,4,5,6,7,8,9,10].

Inulin-type fructans represent a class of non-digestible carbohydrates composed mainly of linear β-(2 → 1)-linked fructose units terminated by a glucose moiety, with a degree of polymerization ranging typically from 2 to 60 units. Due to the absence of endogenous inulinase enzymes in the human digestive system, inulin resists hydrolysis in the upper gastrointestinal tract and is selectively fermented by beneficial microbiota in the colon, particularly *Bifidobacterium* spp. and *Lactobacillus* spp., producing short-chain fatty acids (SCFAs) such as acetate, propionate, and butyrate, which are associated with anti-inflammatory, immunomodulatory and metabolic regulatory effects [1,11,12,13,14,15].

Polyphenols, a diverse group of secondary metabolites including phenolic acids, flavonoids, stilbenes and tannins, exhibit strong antioxidant activity due to their ability to donate hydrogen atoms or electrons and stabilize reactive oxygen species (ROS). Numerous studies have demonstrated that polyphenols contribute to the prevention of oxidative stress-related disorders including cardiovascular diseases, diabetes, cancer and neurodegenerative diseases, through modulation of cellular signaling pathways and regulation of inflammatory mediators [2,3,6,8,9,10,16,17].

Recent research highlights that the biological activity of polyphenols is strongly influenced by their interactions with macromolecules such as dietary fibers, proteins and polysaccharides present in plant matrices. Cell wall polysaccharides may interact with phenolic compounds through hydrogen bonding, hydrophobic interactions, and covalent crosslinking mechanisms, influencing the extractability, stability and bioavailability of antioxidants. These interactions may lead to the formation of non-extractable polyphenols (NEPPs), representing an important fraction of dietary antioxidants reaching the colon, where they are metabolized by microbiota into bioactive metabolites with systemic effects [2,18].

Several medicinal plants rich in inulin-type fructans are also important sources of phenolic antioxidants. For example, *Cichorium intybus* roots contain fructooligosaccharides, chlorogenic acid derivatives, and flavonoids contributing to prebiotic and antioxidant activity. Similarly, *Helianthus tuberosus* tubers represent a valuable source of inulin and polyphenols, demonstrating correlations between inulin content and antioxidant enzymatic parameters such as catalase (CAT), superoxide dismutase (SOD) and ferric-reducing antioxidant power (FRAP). Other inulin-rich species such as *Smallanthus sonchifolius* (yacon) contain caffeic acid derivatives and fructooligosaccharides with demonstrated antioxidant potential [19,20,21,22,23].

Beyond their individual bioactivities, polysaccharide–polyphenol complexes represent an emerging area of research due to their potential to modulate physicochemical properties of plant extracts, improve the stability of bioactive compounds, and enhance antioxidant efficiency. Polyphenol–polysaccharide conjugates isolated from plant matrices demonstrate increased radical scavenging capacity, metal-chelating ability, and modulation of lipid metabolism biomarkers. Furthermore, interactions between phenolic compounds, proteins, and carbohydrates influence structural and functional properties of food matrices, affecting bioavailability and biological activity of antioxidants [2,24].

Technological processing conditions may also influence the stability of polyphenols in the presence of inulin. Studies have demonstrated that inulin acts as a carrier matrix protecting anthocyanins and phenolic acids during drying processes, improving retention of bioactive compounds in functional food formulations. Moreover, encapsulation strategies using inulin or other polysaccharides have been shown to enhance total phenolic content and antioxidant capacity in medicinal plant extracts used for biofunctional food preparation [25,26,27,28].

The growing interest in functional foods enriched with dietary fibers and polyphenols is supported by increasing evidence that such combinations contribute to modulation of gut microbiota composition and improvement of metabolic health. Inulin-enriched formulations have been shown to improve mineral absorption, lipid metabolism, and glycemic control, supporting their potential role in prevention of metabolic disorders. *Jerusalem artichoke* tubers have been proposed as valuable sources of functional ingredients due to their high content of inulin-type fructans and polyphenols with potential benefits in diabetes prevention and metabolic regulation [1,3,4,5,7,22,29,30,31,32].

Understanding the interactions between inulin-type fructans and polyphenols is therefore essential for the development of innovative phytopharmaceuticals, nutraceuticals, and functional foods with enhanced biological activity. The synergistic mechanisms between polysaccharides and phenolic compounds may provide new perspectives for designing advanced formulations targeting oxidative stress, inflammatory disorders, and microbiota-related diseases [2,3,4,12,14,15,28,33].

The aim of the present review is to provide an updated overview of the structural characteristics of inulin, sources of inulin-rich medicinal plants, molecular interactions between polysaccharides and polyphenols, and their implications for antioxidant activity, bioavailability and functional applications in phytotherapy and nutraceutical science. Several reviews have independently addressed either the prebiotic effects of inulin-type fructans or the antioxidant properties of plant polyphenols. However, limited attention has been given to the physicochemical and biological interactions between these two classes of compounds and how such interactions influence antioxidant stability, bioavailability and functional food performance.

## 2. Review Methodology

We conducted a narrative literature review aiming to provide an integrated overview of the interactions between inulin-type fructans and plant polyphenols, with particular emphasis on antioxidant activity, bioavailability, gut microbiota modulation, and functional food applications.

The literature search was performed using the electronic databases PubMed, Scopus, Web of Science and Google Scholar. Publications published between January 2000 and March 2026 were considered. The search strategy included combinations of the following keywords: “inulin”, “inulin-type fructans”, “fructooligosaccharides”, “polyphenols”, “phenolic compounds”, “antioxidant activity”, “bioavailability”, “gut microbiota”, “functional foods”, “nutraceuticals”, “encapsulation”, and “prebiotics”.

The inclusion criteria comprised original research articles, review papers and book chapters reporting information on the chemical structure, extraction, physicochemical interactions, antioxidant properties, bioavailability or technological applications of inulin and polyphenol-containing systems. Studies addressing both plant-derived and food-derived matrices were considered. Human studies, animal experiments, in vitro investigations, and formulation studies were included when relevant to the objectives of the review.

The exclusion criteria included duplicate records, conference abstracts without full-text availability, non-English publications, studies lacking sufficient methodological details, and publications not directly related to inulin–polyphenol interactions or their functional implications.

The retrieved literature was screened according to title, abstract and full-text relevance. Particular attention was given to studies reporting experimental evidence regarding molecular interaction mechanisms, antioxidant activity measurements, bioavailability outcomes, and functional food applications. Evidence was categorized according to study type, including in vitro investigations, formulation and processing studies, animal experiments, and human intervention studies whenever available.

Based on the selected literature, this review addresses the following research questions:

1. What are the main molecular mechanisms governing interactions between inulin-type fructans and plant polyphenols?
2. How do these interactions influence the antioxidant activity and stability of phenolic compounds?
3. What is the current evidence regarding the effects of inulin–polyphenol systems on bioavailability and gut microbiota modulation?
4. How can these interactions be exploited in the development of functional foods and nutraceutical formulations?

The novelty of this review lies in integrating evidence from food chemistry, antioxidant research, gastrointestinal bioaccessibility, and formulation science to provide a comprehensive perspective on the synergistic relationship between inulin-type fructans and plant polyphenols.

## 3. Chemical Structure and Sources of Inulin and Extraction of Inulin-Type Fructans

Inulin represents one of the most important storage polysaccharides in higher plants, particularly within species belonging to the Asteraceae family. Structurally, inulin is a linear fructan consisting of β-(2 → 1)-linked fructofuranosyl units typically terminated by a glucose moiety derived from sucrose. The degree of polymerization (DP) generally ranges from 2 to 60 units, depending on plant species, physiological stage of development, environmental conditions, and extraction methodology. The physicochemical properties of inulin are strongly influenced by the DP distribution. Short-chain fructooligosaccharides (FOS) exhibit higher solubility and sweetness, whereas long-chain inulin displays increased viscosity, gel-forming capacity and fat mimetic properties, making it suitable for functional food formulations and pharmaceutical applications. The presence of multiple hydroxyl groups enables hydrogen bonding and interaction with water molecules, proteins and phenolic compounds, contributing to stabilization of bioactive compounds in plant matrices [1,4,11,12,13,14,15,34].

In medicinal and aromatic plants, inulin plays an important physiological role as carbohydrate reserve and osmotic regulator, particularly in species adapted to temperate climates. The distribution of inulin in plant tissues is influenced by environmental stress factors such as drought, temperature variation and soil composition, which may affect both quantitative and qualitative composition of fructans [34,35].

**Figure 1:**
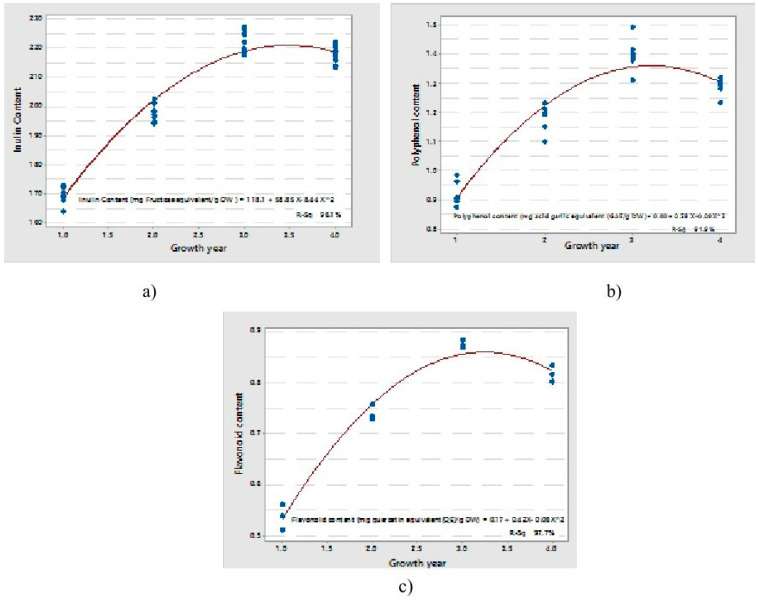
The correlation between the content of inulin (**a**), polyphenol (**b**), and flavonoid (**c**) according to the growth time [35].

Major plant sources of inulin include roots, rhizomes and tubers of species such as *Cichorium intybus* (chicory), *Helianthus tuberosus* (*Jerusalem artichoke*), *Smallanthus sonchifolius* (yacon), *Taraxacum officinale* (dandelion), and *Inula helenium*. Chicory root represents one of the most important commercial sources of inulin, containing up to 70% inulin in dry weight, depending on cultivar and harvesting period. *Jerusalem artichoke* tubers may contain up to 80% inulin-type fructans in dry matter, representing a valuable raw material for functional foods and nutraceutical formulations [20,21,22,23,34] (Figure 1,Table 1).

Yacon (*Smallanthus sonchifolius*) has also attracted considerable interest due to its high content of fructooligosaccharides and phenolic compounds, including caffeic acid derivatives with antioxidant properties. These compounds contribute to the biological activity of plant extracts through synergistic effects between polysaccharides and phenolic antioxidants. The content of inulin varies significantly depending on botanical origin, plant maturity stage, and environmental conditions. Studies have demonstrated that growth time influences accumulation of polysaccharides and polyphenols, with maximum concentrations observed during specific phenological stages [23,35].

Extraction techniques play an essential role in determining the yield, purity, and structural integrity of inulin. Conventional extraction methods typically involve hot water extraction at temperatures between 60 and 80 °C, followed by purification through precipitation, filtration, or membrane separation processes. Extraction efficiency depends on parameters such as solvent-to-solid ratio, extraction time, temperature and pH, which influence solubility and degradation of fructan chains [34].

Advanced extraction technologies including ultrasound-assisted extraction, enzyme-assisted extraction and pressure-assisted extraction have demonstrated improved recovery of inulin and polyphenols from plant matrices. Pectinase-assisted extraction methods have been shown to increase release of intracellular polysaccharides and phenolic compounds from chicory roots, improving biological activity of extracts [24,30].

From a technological perspective, inulin is widely used as a functional ingredient due to its ability to improve texture, stability, and mouthfeel in food formulations. It may act as a fat replacer, sugar substitute, or encapsulating agent for sensitive bioactive compounds. In dairy products, bakery formulations, and nutraceutical powders, inulin contributes to improved rheological properties and enhanced stability of polyphenols during storage and processing [4,11,12,15,25,28,29].

**Figure 2:**
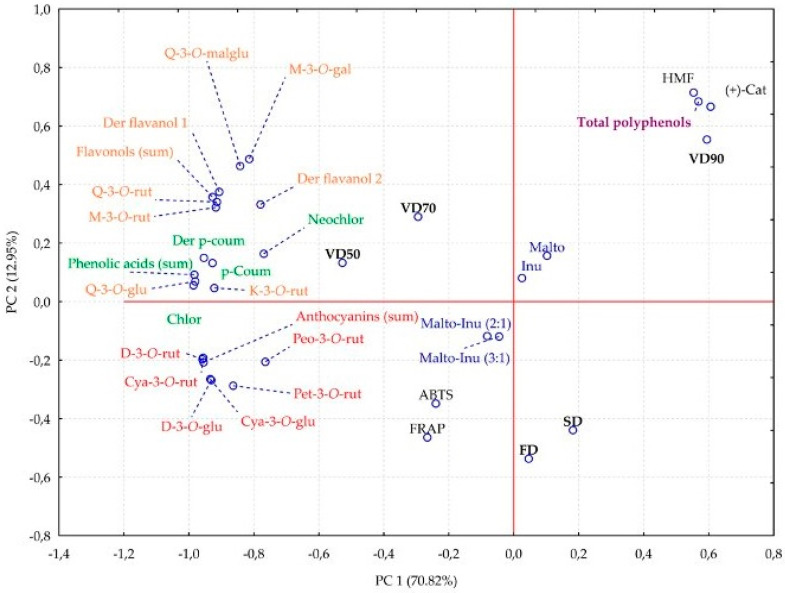
The principal component analysis (PCA) for drying methods (FD—freeze-drying; VD—vacuum-drying at 50 °C, 70 °C, and 90 °C; SD—spray-drying), carriers (Malto—maltodextrin; Inu—inulin; Malto-Inu (2:1 and 3:1)—Maltodextrin–inulin mixed in the proportions of 2:1 and 3:1), identified compounds (HMF—hydroxymethyl-L-furfural; (+)-cat—catechin; D-3-O-rut—delphinidin-3-O-rutinoside; Cya-3-O-rut—anidin-3-O-rutinoside; D-3-O-glu—delphinidin-3-O-glucoside; Cya-3-O-glu—cyanidin-3-O-glucoside; Pet-3-O-glu—petunidin-3-O-glucoside; Peo-3-O-rut—peonidin-3-O-rutinoside; Chlor—chlorogenic acid; p-Coum—p-coumaric acid; Der p-coum—derivative of pcoumaric acid; Neochlor—neochlorogenic acid; M-3-O-rut—myricetin-3-O-rutinoside; Q-3-O-rut—quercetin-3-O-rutinoside; Q-3-O-malglu—quercetin-3-O-malonylglucoside; M-3-O-gal—myricetin-3-O-galactoside; Der flavonol 1, 2—derivative of flavonol 1 and 2), and antioxidant capacity (ABTS—Trolox equivalent antioxidant capacity by ABTS^•+^; FRAP—ferric-reducing antioxidant potential) [25].

Furthermore, the structural characteristics of inulin facilitate formation of supramolecular complexes with polyphenols, influencing antioxidant activity and bioavailability. Hydrogen bonding between hydroxyl groups of fructans and phenolic compounds may stabilize antioxidant molecules and reduce degradation during thermal processing or gastrointestinal digestion [2,25] (Figure 2).

Due to these physicochemical and biological properties, inulin-type fructans represent promising biomolecules for development of innovative phytopharmaceutical formulations and functional foods targeting oxidative stress-related disorders, metabolic diseases, and gut microbiota dysbiosis [3,4,11,12,14,15,28] (Table 2).

## 4. Molecular Interactions Between Inulin and Polyphenols

Interactions between polysaccharides and polyphenols represent a complex physicochemical phenomenon governed by molecular structure, degree of polymerization (DP), spatial conformation, and the presence of functional groups capable of forming intermolecular bonds. Inulin-type fructans contain multiple hydroxyl groups able to participate in hydrogen bonding interactions with phenolic compounds, influencing stability, solubility and bioavailability of antioxidants in plant matrices [2,18].

Polyphenols possess aromatic rings substituted with hydroxyl groups capable of acting both as hydrogen donors and electron donors, contributing to radical scavenging activity. The interaction between inulin and polyphenols may involve hydrogen bonding between hydroxyl groups of fructan chains and phenolic moieties, hydrophobic interactions between aromatic rings and carbohydrate backbone, as well as weak van der Waals forces that contribute to formation of supramolecular complexes. These interactions influence physicochemical properties of plant extracts, including viscosity, stability and resistance to degradation during processing and storage [2,18].

The degree of polymerization of inulin plays an important role in modulating molecular interactions. Long-chain inulin molecules exhibit higher capacity to entrap phenolic compounds within their three-dimensional network, improving protection against thermal degradation and oxidative reactions. Short-chain fructooligosaccharides (FOS), due to higher solubility, may facilitate release and transport of phenolic compounds in aqueous environments, influencing bioaccessibility during digestion [2,4,12,25,27].

Cell wall polysaccharides present in medicinal and aromatic plants may form complexes with polyphenols during extraction, drying or thermal processing. Such interactions influence extractability and antioxidant activity of phenolic compounds, often leading to formation of bound or non-extractable polyphenols (NEPPs), representing an important fraction of dietary antioxidants reaching the colon. NEPPs are gradually released through enzymatic degradation mediated by gut microbiota, producing bioactive metabolites that contribute to systemic antioxidant activity [2,3].

Recent studies demonstrate that polyphenol–polysaccharide conjugates exhibit improved radical scavenging capacity and metal chelation properties compared to free phenolic compounds. These conjugates may also influence lipid metabolism and inflammatory pathways through modulation of cellular signaling mechanisms. The formation of such complexes depends on molecular weight, solubility and structural flexibility of polysaccharides, as well as on the number and position of hydroxyl groups in phenolic compounds [24].

Processing conditions such as temperature, pH, and drying technique significantly influence formation of polysaccharide–polyphenol complexes. Studies on blackcurrant juice powders demonstrated that inulin acts as a protective carrier for anthocyanins and flavonols, improving retention of phenolic compounds during spray-drying and vacuum-drying processes. Similarly, incorporation of inulin into plant extracts has been shown to enhance total phenolic content (TPC) and antioxidant activity, suggesting that polysaccharides may stabilize polyphenolic structures during processing [25,26].

In the gastrointestinal tract, interactions between inulin and polyphenols influence digestion kinetics and metabolic transformation of phenolic compounds. Inulin resists digestion in the upper gastrointestinal tract and undergoes fermentation in the colon, producing short-chain fatty acids (SCFAs) that contribute to modulation of gut microbiota composition and intestinal barrier function. Polyphenols bound to polysaccharides may reach the colon intact, where enzymatic hydrolysis releases bioactive metabolites capable of exerting antioxidant and anti-inflammatory effects [1,5,6,7,8,16,17,22,30,33,40,41].

Furthermore, supramolecular organization of polysaccharide–polyphenol complexes may influence encapsulation and controlled release mechanisms in functional food matrices and phytopharmaceutical formulations. The presence of inulin may improve the stability of phenolic compounds against oxidation, light exposure and enzymatic degradation, contributing to enhanced shelf life of nutraceutical products [4,12,25,26,27,28].

Understanding the molecular interactions between inulin-type fructans and polyphenols is essential for development of innovative functional foods and phytotherapeutic formulations targeting oxidative stress, metabolic disorders, and gut microbiota dysbiosis. The synergistic relationship between dietary fibers and phenolic antioxidants represents an emerging research field with significant potential for applications in pharmacognosy, nutraceutical science, and functional product design [2,3,4,5,6,7,8,33,41].

**Figure 3:**
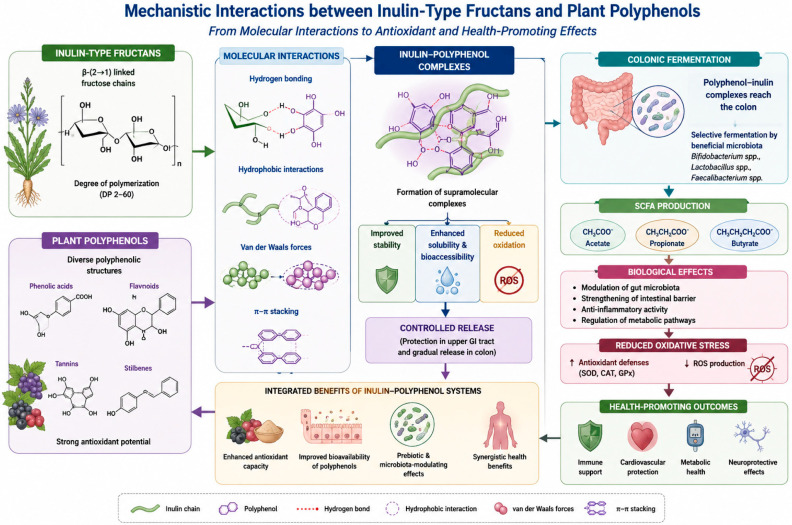
Mechanistic interactions between inulin-type fructans and plant polyphenols and their implications for antioxidant activity, bioavailability, and health-promoting effects. Created by the authors based on the literature reviewed in this article.

The proposed mechanisms governing the synergistic relationship between inulin-type fructans and plant polyphenols are summarized in Figure 3. These interactions influence molecular stability, gastrointestinal bioaccessibility, and microbiota-mediated antioxidant effects, supporting the development of functional foods and nutraceutical formulations with enhanced biological activity (Figure 3).

## 5. Influence of Inulin on Antioxidant Activity

Recent experimental studies highlight the important role of inulin-type fructans in modulating antioxidant activity of plant extracts and functional food matrices. Although inulin itself is not classified as a primary antioxidant molecule, growing evidence suggests that it contributes indirectly to antioxidant effects through stabilization of polyphenols, modulation of gut microbiota metabolism, and improvement of bioavailability of phenolic compounds [5,8,19,25,26,31].

The antioxidant activity of plant matrices containing inulin is commonly evaluated using spectrophotometric assays such as DPPH, ABTS and FRAP, as well as enzymatic antioxidant markers including catalase (CAT), superoxide dismutase (SOD), and peroxidase (POD). These parameters provide important information regarding the capacity of bioactive compounds to neutralize reactive oxygen species (ROS) and reduce oxidative stress [19,25,26,35].

Studies on *Helianthus tuberosus* (*Jerusalem artichoke*), one of the richest natural sources of inulin-type fructans, demonstrated significant correlations between inulin concentration and antioxidant parameters including FRAP values and radical scavenging activity. Increased inulin content was associated with higher activity of antioxidant enzymes such as catalase (CAT) and superoxide dismutase (SOD), suggesting that fructans may contribute to protection of phenolic compounds against oxidative degradation. These findings support the hypothesis that polysaccharide matrices may influence the stability of polyphenols and modulate their antioxidant potential [19,22].

Similar results were observed in studies investigating the influence of growth stage on bioactive compound accumulation in medicinal plants. In *Codonopsis javanica*, variations in harvesting time significantly affected concentrations of inulin, total polyphenols and flavonoids, with corresponding changes in antioxidant capacity measured by ABTS and FRAP assays. These results demonstrate that physiological factors such as plant maturity stage may influence biosynthesis and accumulation of polysaccharides and phenolic compounds, affecting overall antioxidant activity [35].

In addition to plant physiology factors, technological processing conditions also influence antioxidant activity of inulin-containing matrices. Studies investigating the effect of drying processes on blackcurrant juice powders demonstrated that inulin acts as a protective carrier for anthocyanins and flavonols, improving retention of phenolic compounds and reducing degradation during thermal treatment. The protective effect of inulin may be attributed to formation of hydrogen bonds and encapsulation mechanisms that reduce exposure of phenolic compounds to oxygen, heat, and light [25].

Research on biofunctional food formulations enriched with medicinal plant extracts also demonstrated that addition of inulin increases total phenolic content (TPC) and radical scavenging activity. Encapsulation of polyphenols using inulin or other polysaccharides contributes to stabilization of phenolic compounds and improved antioxidant capacity in food matrices. These findings confirm the role of inulin as functional ingredient capable of improving stability of antioxidants in complex matrices [26] (Table 3).

The mechanisms responsible for the influence of inulin on antioxidant activity may include both physicochemical and biological processes. Physicochemically, inulin contributes to stabilization of phenolic compounds through hydrogen bonding interactions and reduction of molecular mobility, decreasing susceptibility to oxidation. Biologically, fermentation of inulin by intestinal microbiota produces short-chain fatty acids (SCFAs) which modulate inflammatory processes and improve intestinal barrier function, indirectly contributing to reduction of oxidative stress [1,2,3,8,14,41].

Furthermore, synergistic interactions between inulin and polyphenols may influence cellular antioxidant defense mechanisms through modulation of signaling pathways involved in oxidative stress response. Polyphenol metabolites released during fermentation may act as signaling molecules influencing gene expression related to antioxidant enzymes [3].

Overall, available evidence suggests that inulin-rich plant matrices may enhance antioxidant potential of phenolic compounds through stabilization, controlled release, and modulation of gut microbiota metabolism. These properties support the use of inulin as functional ingredient in the development of nutraceuticals, phytopharmaceutical formulations, and functional foods targeting oxidative stress-related disorders [3,5,8,19,25,26,31,41].

## 6. Functional Food Applications of Inulin–Polyphenol Systems

Inulin-type fructans are widely used as functional ingredients in food technology due to their unique physicochemical and physiological properties. As soluble dietary fibers, inulin molecules exhibit high water-binding capacity, gel-forming ability, and fat mimetic behavior, making them suitable for development of low-calorie foods with improved texture and stability. The ability of inulin to form microcrystalline structures in aqueous environments contributes to modification of rheological properties and improvement of mouthfeel, particularly in dairy products, bakery formulations, and nutraceutical matrices [4,11,12,15,28].

One of the most important technological applications of inulin is its use as a fat replacer in reduced-calorie foods. Due to its ability to form creamy textures and stabilize emulsions, inulin is frequently incorporated into yogurt, cheese, spreads, and desserts. In yogurt formulations, addition of inulin improves viscosity and reduces syneresis, contributing to improved stability and sensory acceptability of the final product. These properties are attributed to the formation of a three-dimensional network capable of retaining water and stabilizing protein structures [29].

Inulin is also widely used as a carrier and encapsulating matrix for polyphenols and other sensitive bioactive compounds. Studies on fruit juice powders demonstrated that inulin protects anthocyanins and flavonols during drying processes, improving retention of phenolic compounds and antioxidant capacity. The encapsulation ability of inulin is associated with formation of hydrogen bonds between fructan chains and phenolic hydroxyl groups, reducing exposure of polyphenols to oxygen and heat during technological processing [25,27,28].

Functional food formulations enriched with plant extracts rich in polyphenols and polysaccharides have demonstrated enhanced antioxidant activity and improved stability of bioactive compounds. Research investigating medicinal aromatic plant extracts showed that addition of inulin or other polysaccharides increased total phenolic content (TPC) and radical scavenging activity, supporting the development of biofunctional foods with improved health-promoting properties [26].

From a nutritional perspective, inulin exerts prebiotic effects by selectively stimulating growth of beneficial intestinal bacteria such as *Bifidobacterium* and *Lactobacillus* species. Fermentation of inulin in the colon produces short-chain fatty acids (SCFAs), including acetate, propionate and butyrate, which contribute to modulation of intestinal pH, improvement of epithelial barrier integrity and reduction of inflammatory processes. These physiological effects are associated with improved gastrointestinal health and modulation of immune responses [1,3,13,14,30,32].

Functional foods enriched with inulin and polyphenols have demonstrated potential benefits in prevention and management of metabolic disorders such as obesity, insulin resistance, and metabolic syndrome. The presence of soluble dietary fibers contributes to reduction of glycemic response and improvement of lipid metabolism, while polyphenols exert antioxidant and anti-inflammatory effects through modulation of cellular signaling pathways. *Jerusalem artichoke*-derived inulin has been proposed as functional ingredient with potential application in diabetic diets due to its ability to act as sugar substitute without significantly influencing blood glucose levels [1,4,22].

In addition to metabolic benefits, the combination of inulin and polyphenols contributes to a reduction in oxidative stress through synergistic antioxidant mechanisms. Polyphenols act as direct radical scavengers, while inulin improves stability and bioavailability of these compounds, facilitating sustained release in the gastrointestinal tract. Such synergistic effects support development of innovative functional foods targeting chronic inflammatory diseases and oxidative stress-related disorders. Recent advances in food formulation technologies have enabled the development of symbiotic products combining prebiotics such as inulin with probiotic microorganisms and plant-derived antioxidants. These products demonstrate improved stability, enhanced antioxidant capacity, and increased health-promoting potential. The integration of medicinal plant extracts into functional food matrices represents an important direction in nutraceutical research and supports the development of personalized nutrition strategies [2,3,4,5,6,7,8,25,31,32,33].

Overall, the multifunctional role of inulin as texture modifier, stabilizing agent, encapsulating matrix and prebiotic compound makes it a valuable ingredient in development of functional foods enriched with polyphenols. The synergistic interactions between polysaccharides and phenolic compounds contribute to improved antioxidant activity, enhanced bioavailability and modulation of gut microbiota, supporting the design of innovative food products with health-promoting properties [4,11,25,26,28,32].

## 7. Pharmacological and Nutraceutical Implications of Inulin–Polyphenol Systems

Inulin-type fructans represent one of the most extensively studied classes of prebiotic polysaccharides due to their capacity to modulate gut microbiota composition and influence multiple physiological pathways associated with metabolic health and oxidative stress. Unlike digestible carbohydrates, inulin resists enzymatic hydrolysis in the upper gastrointestinal tract and reaches the colon largely intact, where it undergoes fermentation by beneficial microbial populations such as *Bifidobacterium* spp. and *Lactobacillus* spp. This selective fermentation process contributes to modulation of intestinal microbiota balance and inhibition of pathogenic bacterial strains involved in inflammatory disorders [1,3,13,14,15].

Fermentation of inulin leads to production of short-chain fatty acids (SCFAs), including acetate, propionate and butyrate, which play important roles in maintaining intestinal homeostasis, regulating epithelial barrier function and modulating immune responses. Butyrate, in particular, has been associated with anti-inflammatory and anticarcinogenic properties due to its capacity to regulate gene expression involved in cell proliferation and apoptosis. SCFAs also contribute to a reduction in intestinal pH, creating an unfavorable environment for pathogenic microorganisms and promoting growth of beneficial bacteria [1,3].

In addition to microbiota modulation, inulin has been shown to influence mineral absorption, particularly calcium and magnesium. Increased solubility of minerals in acidic intestinal environment created by SCFA production contributes to improved bioavailability and enhanced intestinal uptake. These effects are particularly relevant in prevention of osteoporosis and metabolic disorders associated with mineral deficiencies [1].

The influence of inulin on lipid metabolism represents another important nutraceutical property. Studies have demonstrated that inulin supplementation may contribute to reduction of serum triglycerides and cholesterol levels through modulation of hepatic lipid metabolism and regulation of genes involved in fatty acid synthesis. The presence of soluble dietary fibers may also influence satiety mechanisms and glycemic response, supporting their role in prevention of obesity and metabolic syndrome [1,14,15,24].

Recent studies indicate that the combination of inulin-type fructans with plant polyphenols may exert synergistic pharmacological effects due to complementary mechanisms of action. Polyphenols act as direct antioxidants capable of scavenging reactive oxygen species (ROS), while inulin contributes to stabilization and controlled release of phenolic compounds in the gastrointestinal tract. This combination may enhance bioavailability of phenolic metabolites and prolong their biological activity [2,3,4,5,6,7,8,25,31,41].

Polyphenol metabolites produced through microbiota-mediated biotransformation may exert systemic antioxidant and anti-inflammatory effects. Such metabolites have been associated with modulation of cellular signaling pathways involved in oxidative stress response, including NF-κB, Nrf2 and MAPK pathways. The interaction between dietary fibers and polyphenols may therefore influence gene expression patterns associated with inflammatory processes and immune regulation [3,8,16,17,41,42,43,44].

Evidence suggests that inulin-rich plant matrices may contribute to protection against colon carcinogenesis through modulation of microbiota composition, production of SCFAs, and reduction of oxidative stress in intestinal epithelial cells. The protective effect of butyrate on colonocytes has been associated with inhibition of tumor cell proliferation and induction of apoptosis in abnormal cells. Polyphenols further enhance these effects through antioxidant activity and modulation of inflammatory mediators [1,3].

In nutraceutical formulations, inulin is frequently used as a carrier matrix for encapsulation of polyphenols, vitamins, and other bioactive compounds. Encapsulation technologies improve the stability of phenolic compounds and allow controlled release in specific segments of the gastrointestinal tract. This strategy contributes to increased efficacy of plant extracts and improved therapeutic potential of phytopharmaceutical formulations [4,12,25,26,27,28].

The combination of prebiotic polysaccharides with antioxidant polyphenols represents a promising strategy for the development of innovative nutraceuticals targeting oxidative stress-related diseases, metabolic disorders, and inflammatory conditions. Such formulations may contribute to modulation of microbiota composition, improvement of intestinal barrier function, and enhancement of antioxidant defense mechanisms [3,4,5,6,7,8,14,31,33].

From a pharmacognostic perspective, medicinal plants rich in inulin and polyphenols represent valuable sources of bioactive compounds with potential applications in phytotherapy. The synergistic relationship between dietary fibers and phenolic antioxidants provides new perspectives for the development of functional ingredients and plant-based formulations with enhanced biological activity [20,21,22,23].

Overall, the pharmacological and nutraceutical implications of inulin–polyphenol systems highlight the importance of integrating polysaccharide-based prebiotics with plant-derived antioxidants in the development of functional foods and phytopharmaceuticals targeting chronic diseases associated with oxidative stress and inflammation [1,2,3,4,14,15,33,41].

## 8. Influence of Inulin on Stability and Retention of Polyphenols

Stability of polyphenolic compounds represents one of the major challenges in development of functional foods and phytopharmaceutical formulations, due to their susceptibility to oxidation, thermal degradation, enzymatic hydrolysis, and photochemical instability. Polyphenols such as anthocyanins, flavonols, and phenolic acids are particularly sensitive to environmental factors including temperature, oxygen exposure, pH variations, and light irradiation. Recent studies indicate that polysaccharide matrices such as inulin may significantly improve stability of polyphenols through formation of protective supramolecular structures [9,25,26,27,41].

Inulin acts as a carrier matrix capable of interacting with phenolic compounds through hydrogen bonding and steric stabilization mechanisms. These interactions reduce molecular mobility and limit exposure of reactive hydroxyl groups to oxidative conditions. Studies investigating blackcurrant (*Ribes nigrum*) juice powders demonstrated that addition of inulin improved retention of anthocyanins and flavonols during drying processes, particularly under vacuum-drying conditions at moderate temperatures. The protective effect of inulin was associated with reduced degradation of phenolic compounds and improved color stability of the final product [26,27].

Encapsulation techniques using polysaccharides such as inulin and gum arabic have demonstrated increased total phenolic content (TPC) and enhanced radical scavenging activity in medicinal plant extracts. Encapsulation protects polyphenols against oxidation and enzymatic degradation, improving stability during storage and gastrointestinal digestion. The ability of inulin to form amorphous or microcrystalline matrices contributes to the controlled release of bioactive compounds and the increased shelf life of nutraceutical products [26,27,28].

Processing conditions such as spray-drying, freeze-drying and vacuum-drying influence the stability of polyphenols in the presence of inulin. The formation of glassy matrices reduces diffusion of oxygen and limits degradation reactions. Moreover, interactions between polysaccharides and phenolic compounds may reduce formation of degradation products such as hydroxymethylfurfural (HMF), preserving the biological activity of plant extracts [25].

Inulin has also demonstrated protective effects against the degradation of phenolic compounds during the storage of functional beverages and nutraceutical powders. The presence of polysaccharide matrices reduces oxidation kinetics and stabilizes phenolic hydroxyl groups responsible for antioxidant activity. These properties support the use of inulin as a technological ingredient for the stabilization of polyphenols in complex plant-based formulations [25,26,27,28,41].

## 9. Bioavailability and Gut Microbiota Modulation

Bioavailability of polyphenols represents a critical factor influencing biological activity of plant-derived antioxidants. Many polyphenols occur in plant matrices bound to cell wall polysaccharides, proteins or lignin, forming complexes that limit absorption in the upper gastrointestinal tract. Inulin-type fructans contribute to modulation of bioavailability through their prebiotic effects and influence on intestinal microbiota metabolism [2,3,4,5,6,7,8,9,16,17,41,44].

Fermentation of inulin by intestinal microbiota results in production of short-chain fatty acids (SCFAs) including acetate, propionate and butyrate, compounds associated with anti-inflammatory activity and improvement of intestinal barrier integrity. SCFAs regulate expression of genes involved in immune response, oxidative stress regulation, and epithelial cell differentiation. Butyrate has been shown to influence histone acetylation processes and modulate gene expression associated with inflammatory signaling pathways [1,3].

Polyphenols undergo extensive biotransformation mediated by intestinal microbiota, producing metabolites with increased bioactivity and improved absorption. Microbial enzymes such as esterases and glycosidases hydrolyze complex phenolic structures, releasing smaller phenolic acids that are capable of crossing intestinal barrier. Inulin may enhance these processes by stimulating the growth of beneficial bacterial populations such as *Bifidobacterium* spp. and *Lactiplantibacillus* spp., while inhibiting pathogenic microorganisms associated with inflammatory diseases [3,4,5,6,7,8,10,16,17,41,42,43,44].

The interaction between inulin and polyphenols contributes to the concept of the diet–microbiota–antioxidant axis, which describes the relationship between dietary components, microbial metabolism, and antioxidant defense mechanisms. Polyphenol metabolites produced during microbial fermentation may act as signaling molecules regulating oxidative stress response pathways including Nrf2 signaling cascade [3,4,5,6,7,8,41].

Recent studies demonstrate that combined administration of prebiotic fibers and polyphenols contributes to modulation of intestinal permeability and reduction of endotoxin translocation associated with metabolic disorders. Improvement of intestinal barrier function is associated with decreased systemic inflammation and reduced oxidative stress [3,5,30,31,32,33].

## 10. Mechanistic Model of Synergistic Antioxidant Effects

Synergistic antioxidant effects between inulin-type fructans and polyphenols are based on multiple molecular and physiological mechanisms that influence the stability, bioavailability, and biological activity of phenolic compounds. These mechanisms involve both physicochemical interactions occurring in plant matrices and biological processes occurring in gastrointestinal environment [2,3,4,5,6,7,25,41].

### 10.1. Molecular Mechanisms

Hydrogen bonding interactions occur between hydroxyl groups of fructan chains and phenolic hydroxyl groups, contributing to formation of supramolecular complexes. These complexes reduce exposure of polyphenols to oxidative degradation and increase resistance to thermal stress [2].

Hydrophobic interactions between aromatic rings of polyphenols and hydrophobic regions of polysaccharide chains contribute to stabilization of phenolic compounds within plant matrices. Van der Waals forces further contribute to formation of stable supramolecular assemblies capable of protecting antioxidants during processing and digestion [2,18].

Covalent conjugation between phenolic compounds and cell wall polysaccharides may occur through oxidative coupling reactions mediated by enzymatic or non-enzymatic mechanisms. These conjugates contribute to formation of non-extractable polyphenols (NEPPs), representing an important fraction of dietary antioxidants reaching the colon [2,24].

Encapsulation of polyphenols within fiber matrices contributes to protection against oxidation and allows gradual release during digestion, improving persistence of antioxidant activity [12,25,26,27,28].

### 10.2. Physiological Mechanisms

Delayed release of polyphenols in gastrointestinal tract contributes to prolonged antioxidant activity and increased interaction with intestinal microbiota. Inulin fermentation stimulates microbial production of SCFAs, which contribute to modulation of inflammatory pathways and improvement of intestinal barrier function [1,2,3,4,5,6,7,8,13,14,30,33].

Increased SCFA production contributes to regulation of lipid metabolism and modulation of immune response through activation of G-protein-coupled receptors involved in inflammatory processes [1,3].

Synergistic interactions between inulin and polyphenols may also influence expression of antioxidant enzymes such as superoxide dismutase (SOD) and catalase (CAT), contributing to improved cellular defense mechanisms against oxidative stress [19].

Overall, the combination of polysaccharide-based prebiotics and phenolic antioxidants represents a promising strategy for development of advanced functional foods and phytopharmaceutical formulations with improved biological activity. Understanding these mechanisms provides valuable insights for design of innovative nutraceutical systems targeting oxidative stress, inflammatory diseases, and metabolic disorders [2,3,4,5,6,14,15,25,26,27,28,32,33].

## 11. Research Gaps and Future Perspectives

### 11.1. Lack of Clinical Evidence

Most available studies investigating inulin–polyphenol interactions are based on in vitro experiments or animal models. Additional randomized clinical trials are required to validate their biological effects in humans. Furthermore, the influence of age, dietary habits and gut microbiota composition on treatment outcomes remains insufficiently investigated. Long-term intervention studies are needed to establish clinically relevant dosages and health benefits.

### 11.2. Standardization of Inulin Sources

Variability in botanical origin, extraction procedures and degree of polymerization may significantly influence biological activity and should be standardized.

### 11.3. Advanced Delivery Systems

Future research should focus on encapsulation technologies, nanoformulations, and controlled-release systems capable of improving polyphenol stability and bioavailability.

### 11.4. Personalized Nutrition Approaches

The interaction between inulin-type fructans, polyphenols, and gut microbiota composition suggests potential applications in personalized nutrition and precision nutraceuticals.

### 11.5. Sustainable Functional Food Development

The exploitation of inulin-rich plant resources represents an attractive strategy for developing sustainable functional foods with antioxidant and prebiotic properties.

## 12. Conclusions

The present review highlights the growing scientific evidence supporting the synergistic interactions between inulin-type fructans and plant polyphenols, emphasizing their combined contribution to antioxidant activity, bioavailability, and functional performance in nutraceutical and functional food systems. Although inulin itself does not exhibit strong direct antioxidant capacity, numerous experimental studies demonstrate that inulin-rich matrices enhance the stability, retention, and bioefficacy of phenolic compounds through physicochemical and biological mechanisms [1,2,3,4,5,6,7,8,9,10,14,41].

Molecular interactions between polysaccharides and polyphenols—including hydrogen bonding, hydrophobic interactions, and the formation of supramolecular complexes—significantly influence the extractability, stability and controlled release of antioxidants in plant matrices. The formation of non-extractable polyphenols (NEPPs) associated with dietary fibers contributes to the delayed release of bioactive metabolites in the colon, where microbiota-mediated transformation generates compounds with systemic antioxidant and anti-inflammatory effects [2,3,24].

Experimental data indicate correlations between inulin content and antioxidant parameters measured using DPPH, ABTS and FRAP assays, as well as enzymatic antioxidant markers such as catalase (CAT) and superoxide dismutase (SOD). These findings suggest that inulin plays an indirect but important role in modulating antioxidant defense mechanisms through stabilization of phenolic compounds and interaction with gut microbiota metabolism [19,35].

Technological applications of inulin in functional foods demonstrate its ability to improve the texture, rheological properties, and stability of bioactive compounds. As a fat mimetic agent, encapsulating matrix, and prebiotic ingredient, inulin contributes to the development of innovative formulations with improved sensory properties and enhanced health-promoting potential. Encapsulation strategies using inulin have shown increased retention of anthocyanins, flavonoids, and phenolic acids during drying and storage processes, supporting the role of polysaccharides as protective carriers for sensitive bioactive compounds [4,12,25,26,27,28,29,30,31,32].

The interaction between dietary fibers and polyphenols is strongly linked to modulation of gut microbiota composition and production of short-chain fatty acids (SCFAs), including acetate, propionate and butyrate, compounds involved in regulation of inflammatory pathways, intestinal barrier function, and metabolic homeostasis. The concept of the diet–microbiota–antioxidant axis provides an integrated framework explaining the relationship between plant-derived bioactive compounds, microbial metabolism, and prevention of oxidative stress-related disorders [1,2,3,4,5,6,7,8,16,41,42].

From a pharmacognostic and phytotherapeutic perspective, medicinal and aromatic plants rich in both inulin and polyphenols represent valuable sources of multifunctional bioactive compounds with potential applications in prevention of metabolic disorders, inflammatory diseases and microbiota-related dysbiosis. The synergistic combination of prebiotic polysaccharides and phenolic antioxidants may contribute to development of advanced nutraceutical systems targeting oxidative stress and chronic inflammation [20,21,22,23].

Future research directions should focus on elucidating the structure–activity relationships governing polysaccharide–polyphenol interactions, optimization of extraction and encapsulation technologies, and evaluation of bioavailability using in vivo and clinical models. Standardization of analytical methodologies for the characterization of non-extractable polyphenols and investigation of microbiota-mediated metabolic pathways will further improve understanding of synergistic antioxidant mechanisms [2,3,7,31,33,41].

Overall, the integration of inulin-type fructans with plant polyphenols represents a promising strategy for the development of innovative functional foods and phytopharmaceutical formulations with enhanced biological activity. Understanding the complex interactions between dietary fibers, phenolic compounds, and gut microbiota provides new perspectives for designing nutraceutical products capable of modulating oxidative stress and supporting human health [1,2,3,4,5,6,7,8,9,10,14,25,26,27,28,32,33].

## Figures and Tables

**Table 1:** Major medicinal and food plants rich in inulin-type fructans and representative poly phenols reported in the literature.

Plant Species	Family	Plant Part	Inulin Content (% DW)	Major Polyphenols
*Cichorium intybus* L.	*Asteraceae*	Root	60–70	Chicoric acid, chlorogenic acid, caffeic acid derivatives
*Helianthus tuberosus* L.	*Asteraceae*	Tuber	50–80	Chlorogenic acid, caffeic acid, flavonoids
*Smallanthus sonchifolius* (Poepp.) H.Rob.	*Asteraceae*	Root	40–70	Chlorogenic acid, dicaffeoylquinic acids, caffeic acid derivatives
*Taraxacum officinale* F.H.Wigg.	*Asteraceae*	Root	20–40	Chicoric acid, luteolin derivatives, chlorogenic acid
*Inula helenium* L.	*Asteraceae*	Root	30–45	Chlorogenic acid derivatives, caffeic acid derivatives, flavonoids

Note: Data compiled from references [20,21,22,23,34,35]. Inulin content may vary depending on cultivar, geographical origin, harvesting period and extraction methodology.

**Table 2:** Methods of extracting inulin-type fructans from plant materials and their main characteristics.

Method	Typical Conditions	Advantages	Limitations	Representative References
Hot water extraction	60–80 °C; 30–120 min; water-to-solid ratio 10:1–20:1	Simple, low cost, industrially applicable	Possible degradation of low-DP fructans and co-extraction of impurities	[4,34,36]
Ultrasound-assisted extraction (UAE)	20–40 kHz; 20–60 min; 30–70 °C	Higher extraction yield, reduced extraction time, lower solvent consumption	Equipment cost and possible structural modifications at high intensity	[34,37]
Enzyme-assisted extraction (EAE)	Pectinase, cellulase or hemicellulase; 40–50 °C; pH 4.5–5.5	Improved recovery of inulin and associated polyphenols; mild conditions	Enzyme cost and process optimization required	[24,30,38]
Pressurized liquid extraction (PLE)	50–150 bar; 50–120 °C	Efficient extraction, reduced processing time	Specialized equipment required	[34,39]

Table footnote: DP = degree of polymerization; UAE = ultrasound-assisted extraction; EAE = enzyme-assisted extraction; PLE = pressurized liquid extraction.

**Table 3:** Evidence of antioxidant enhancement.

Study Model	Antioxidant Assay	Main Finding	Ref.
*Jerusalem artichoke*	FRAP, CAT, SOD	Positive correlation between inulin content and antioxidant activity	[19,22]
*Codonopsis javanica*	ABTS, FRAP	Correlation between inulin, polyphenols and antioxidant capacity	[35]
Blackcurrant powder + inulin	ABTS, TPC	Improved retention of polyphenols during drying	[25]
MAP extracts + inulin	DPPH, TPC	Increased antioxidant activity and total phenolic content	[26]

## Data Availability

Data sharing is not applicable to this review article as no new data were created or analyzed.
